# Feasibility of inpatient cardiac rehabilitation after percutaneous mitral valve reconstruction using clipping procedures: a retrospective analysis

**DOI:** 10.1186/s13102-022-00517-y

**Published:** 2022-07-05

**Authors:** Thomas Schmidt, Marek Kowalski, Birna Bjarnason-Wehrens, Frank Ritter, Gerold Mönnig, Nils Reiss

**Affiliations:** 1 Institute for Cardiovascular Research, Schüchtermann-Klinik Bad Rothenfelde, Ulmenallee 5-11, 49214 Bad Rothenfelde, Germany; 2 Department of Preventive and Rehabilitative Sport and Exercise Medicine, Institute for Cardiology and Sports Medicine, German Sports University Cologne, Am Sportpark Müngerdorf 6, 50933 Cologne, Germany

**Keywords:** Mitral regurgitation, Mitraclip, Cardiac rehabilitation, Exercise intervention

## Abstract

**Background:**

To date, no studies on the feasibility or outcomes of cardiac rehabilitation (CR) after percutaneous mitral valve reconstruction using clipping procedures have been published. The aim of this study was to report on our first experiences with this special target group.

**Methods:**

Monocentric retrospective analysis of 27 patients (72 ± 12 years old, 52% female) who underwent multimodal inpatient CR in the first 2 month after MitraClip™ implantation. A six-minute-walking-test, a handgrip-strength-test and the Berg-Balance-Scale was conducted at the beginning and end of CR. Echocardiography was performed to rule out device-related complications.

**Results:**

Adapted inpatient CR started 16 ± 13 days after clipping intervention and lasted 22 ± 4 days. In 4 patients (15%) CR had to be interrupted or aborted prematurely due to cardiac decompensations. All other patients (85%) completed CR period without complications. Six-minute-walking-distance improved from 272 ± 97 to 304 ± 111 m (p < .05) and dependence on rollator walker or walking aids was significantly reduced (p < .05). Results of handgrip-strength-test and Berg-Balance-Scale increased (p < .05). Overall, social-medical and psychological consultations were well received by the patients and no device-related complications occurred during rehabilitation treatments.

**Conclusions:**

The results indicate that an adapted inpatient CR in selected patients after MitraClip™ implantation is feasible. Patients benefited from treatments both at functional and social-medical level and no device-related complications occurred. Larger controlled studies are needed.

## Introduction

**Fig. 1:**
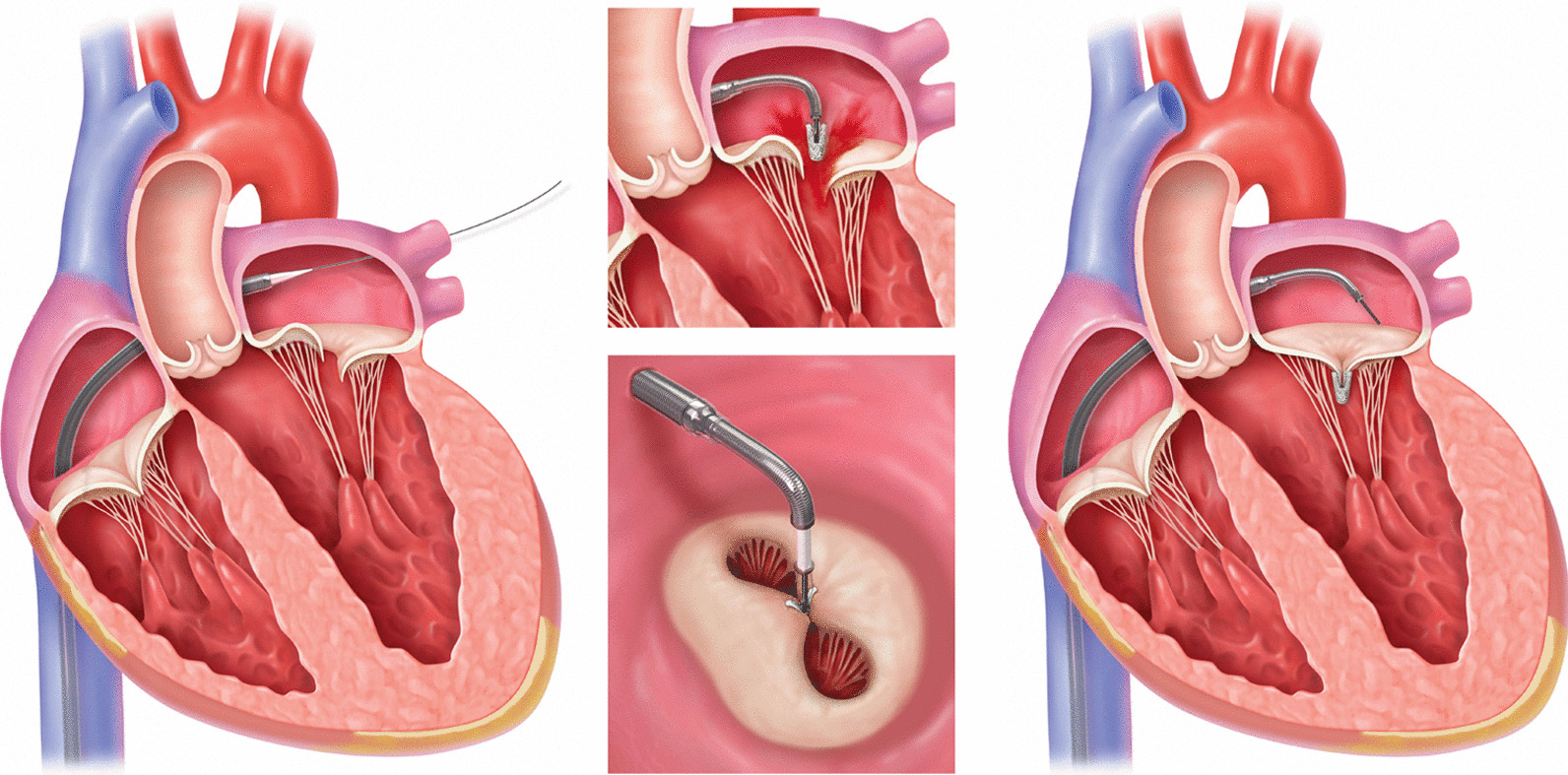
Percutaneous mitral valve reconstruction using the MitraClip™ device. (MitraClip™ is a trademark of Abbott or its related companies. Reproduced with permission of Abbott, © 2022. All rights reserved.)

Valvular heart disease (VHD) is common in elderly patients, and its importance will increase in the coming decades due to demographic change [1, 2]. In the OxVALVE cohort study (2500 individuals, ≥ 65 years), 51% of participants were diagnosed with previously undetected VHD, with 11.3% having moderate or severe manifestations [2]. Data from Germany show that the number of hospital admissions due to VHD has been steadily increasing for many years [3]. The second most common VHD (after aortic sclerosis) is Mitral insufficiency (MI) [2] with frequent occurrence especially in ageing heart failure (HF) patients. According to current guidelines [4, 5], treatment of secondary mitral regurgitation may also be considered via percutaneous mitral valve interventions in patients at high surgical risk. Therefore, clipping procedures (e.g. MitraClip™, Abbott, Fig. 1) have become an established method in recent years [6, 7].

However, despite an increasing number of patients, no studies on the feasibility or outcomes of cardiac rehabilitation (CR)—whether performed in an inpatient or outpatient setting—after MitraClip™ implantation have been published to date, with the exception of one theoretical draft paper [8]. One reason could be that physical rest is often recommended immediately after implantation to prevent dislocation of the clip, and that patients are therefore not prescribed CR.

In our opinion, this is not ideal (at least not as a general precaution) since the often multimorbid patients already suffer from physical inactivity. Common comorbidities are for example arterial hypertension, coronary artery disease, HF, atrial fibrillation, diabetes mellitus or renal failure. Patients are typically old (> 70 years) and many of them had previous cardiac interventions (e.g. coronary bypass grafting) or cardiac decompensations (> 50% within 6 months and > 30% within 21 days) prior to clipping procedure with prolonged hospital stay and physical inactivity periods [9, 10]. Consequently, impaired peripheral adaptive mechanisms and progressive skeletal muscle deconditioning (sarcopenia, frailty) are widely suspected [11]. From our point of view, especially these patients could benefit from multimodal CR in many different ways, since the essential role of physical and rehabilitation medicine (PMR) in the secondary prevention of cardiovascular diseases is evident [12, 13]. This is particularly important given that the long-term results of the COAPT trial show that the six-minute walking distance (6MWD) and its improvement in the first month after MitraClip™ implantation are associated with lower mortality and HF hospitalization, at least for the first 2 years [6, 14].

It is the aim of this study to report our experience with this patient group during inpatient CR program in terms of safety and feasibility in order to start discussions and improve the treatment situation.

## Materials and methods

We conducted a monocentric retrospective analysis of patients who underwent inpatient CR at our hospital within the first 2 months after percutaneous mitral valve intervention between October 2017 and February 2020. The prerequisite for admission and adequate participation in inpatient CR was a stable clinical state and a barthel index of ≥ 65 points.

### Inpatient CR program and diagnostics

The patients received a 3-week multimodal standard inpatient CR program in line with their requirements and condition. The duration of 3 weeks is the typical length for inpatient CR in Germany and is covered by public health insurance.

Therapeutic treatments were agreed with the cardiologist and often included the following:

- aerobic exercise training on a cycle ergometer with monitoring or (if indicated) an assisted cycle therapy (5 times a week/30 min)
- gymnastics in small groups using small equipment like elastic bands or dumbbells (3 times a week/30 min)
- resistance exercise training on weight machines e.g. leg curl or leg extension (2 times a week/30 min)
- outdoor walking training (2 times a week/30 min)
- mechanically guided inhalation training (3 times a week/10 min)
- physiotherapist-guided inspiratory muscle training (3 times a week/20 min)
- arm baths (3 times a week/20 min)
- mud packs (2 times a week, 20 min)
- ergotherapy (3 times a week/30 min)
- individually adapted education (e.g. about HF or VHD)
- socio-medical care
- and also further therapies if indicated.

**Fig. 2:**
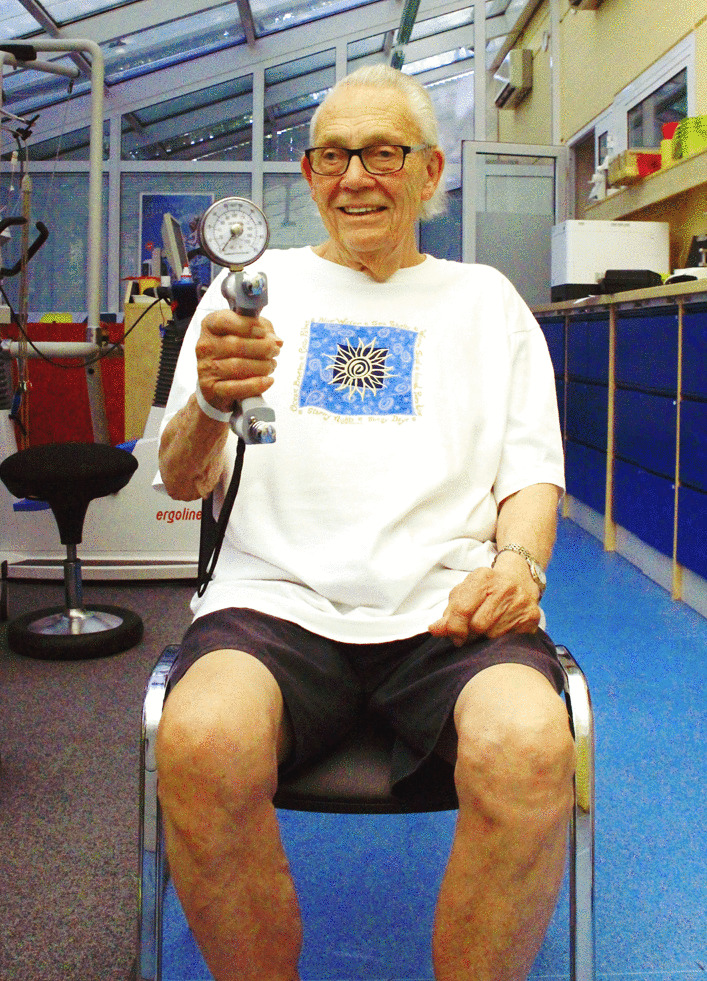
79 years old patient after percutaneous mitral valve reconstruction while performing a handgrip-strength-test during inpatient cardiac rehabilitation

One specific restriction of CR program was that blood pressure peaks should be avoided during treatments (systolic target value ≤ 120 mmHg). For this reason, no maximum cardiopulmonary exercise test (CPET) was performed during CR. Physical exercise units (e.g. aerobic cycle ergometer training or resistance training) were only performed at a low intensity level and incorporated blood pressure monitoring. During the 3-week CR stay, intensity levels were carefully increased depending on the blood pressure values and the perception of the experienced therapists. As part of our standard functional diagnostics, a six-minute-walking-test (6MWT) [15, 16], a handgrip-strength-test [17] (Fig. 2) and the Berg-Balance-Scale (BBS) [18] were performed at the start and end of CR in standardized approach. Likewise, rehabilitation routines included 24-h electrocardiogram (ECG) and 24-h blood pressure measurement. At the end of CR, echocardiography was performed in order to test both MI and the position of the MitraClip™ to rule out possible clip detachments.

### Study population

A total of 27 patients after percutaneous mitral valve interventions via clipping procedures could be included into the study. All patients underwent MitraClip™ implantation and other clipping devices were not observed. Patients were mean 72 ± 12 years old, 52% female and started inpatient CR on average 16 ± 13 days after percutaneous intervention (min 3 days, max 55 days). Before admission, patients achieved a Barthel index of mean 92 ± 9 points (min 70, max 100), indicating sufficient functional independence and the ability to follow inpatient CR program adequately. Detailed patient characteristics are summarized in Table 1.

**Table 1:** Patient characteristics (n = 27)

	${\overline{\text{x}}}$ or n	SD or %
General		
Age (years)	73	± 12
Female (n)	14	52%
Body mass index	26.5	± 4.4
Barthel index (points)	92	± 9
Clinical condition before intervention		
NYHA classification	3.1	± 0.5
Left ventricular ejection fraction (%)	42	± 13
Mitral valve insufficiency (grade)	3.2	± 0.4
Logistic EuroSCORE I (%)	32	± 20
Percutaneous intervention		
1 MitraClip™ (n)	21	78%
2 MitraClips™ (n)	5	18%
3 MitraClips™ (n)	1	4%
ASD occluder needed (n)	4	15%
Clinical condition after intervention and before CR		
Left ventricular ejection fraction (%)	45	± 12
Mitral valve insufficiency (grade)	1.0	± 0.5
Mean gradient mitral valve (mmHg)	3.0	± 1.4
Most common comorbidities at start of CR		
Hypertension (n)	25	93%
Orthopaedic problems (n)	19	70%
Coronary artery disease (n)	16	59%
Atrial fibrillation (n)	16	59%
Renal insufficiency (n)	11	41%
Diabetes mellitus (n)	5	18%
Time		
Implantation to CR (days)	16	± 13
Duration of CR (days)	22	± 4

NYHA, New York Heart Association; ASD, atrial septal defect; CR, cardiac rehabilitation

### Data collection and analyses

Within this evaluation, clinical data from rehabilitation stay and discharge reports from acute hospital stay were examined retrospectively and anonymously. Processing and statistical analysis were performed using the IBM SPSS Statistics software (Version 26, IBM). Comparisons of two nominal variables were conducted using the Fisher’s exact test. For dependent comparisons of mean values, a dependent t-test and/or Wilcoxon rank sum test was used. All statistical comparisons were two-tailed, and the level of significance was set at *alpha* 5%.

According to our local ethics committee (German Sports University Cologne) specific approval for this study was not needed due to the retrospective and anonymous internal single-centre design and informed consent could be waived.

## Results

### Inpatient CR completion

Of the 27 patients admitted, 23 (85%) completed CR without complications. One patient (4%) had to interrupt rehabilitation due to cardiac decompensation, but was able to continue successfully after percutaneous coronary intervention (PCI). In 3 patients (11%) CR had to be stopped in the first week, in two cases due to cardiac decompensation, in one due to cardiac decompensation and renal failure. The following results refer to the 24 patients who completed CR.

### Inpatient CR treatments

Patients were admitted for inpatient CR at an average of 16 ± 13 days after MitraClip™ implantation. The mean CR duration was 22 ± 4 days. In 7 patients (26%) the 3-week CR was extended by one week to meet their needs. Core components of exercise-based CR were individually adapted gymnastics in small groups (100%, 7 ± 2 units), low-intensity aerobic exercise training on a cycle ergometer with monitoring (96%, 11 ± 3 units), low-dose resistance exercises (92%, 6 ± 2 units), mechanically guided inhalation training (96%, 6 ± 2 units) and physiotherapist-guided inspiratory muscle training (50%, 7 ± 2 units). If indicated, especially to improve and stabilize perfusion, patients also received physical therapy, such as mud packs (58%, 5 ± 1 units) or arm baths (54%, 3 ± 2 units). Other core components of CR were individually adapted education and socio-medical care.

### Diagnostic values and outcomes

The 24-h diagnostics showed that during a standard rehabilitation day a mean heart rate of 71 ± 9 bpm and a systolic blood pressure of mean 117 ± 10 mmHg were achieved. The maximum daily values were 100 ± 21 bpm and 142 ± 18 mmHg. Main changes during CR in exercise intensities, functional performance values and device-related echocardiography parameters are summarized in Table 2.

**Table 2:** Changes during inpatient cardiac rehabilitation (CR) in exercise intensities, functional performance values and device-related echocardiographic parameters (n = 24)

	Start of CR Mean ± SD	End of CR Mean ± SD	Changes Mean ± SD	*P* value
Training intensities during CR				
Load during bicycle ergometer training (watts)	18.3 ± 6.3	24.6 ± 10	6.3 ± 6.5	.000***
Weight during leg press (kg)	27.5 ± 5.5	33.1 ± 9.7	5.6 ± 7.5	.006**
Functional performance values				
6MWD (m)	272 ± 97	304 ± 111	32 ± 64	.028*
6MWD (% of predicted)	60 ± 26	67 ± 29	7 ± 14	.033*
6MWD below 300 m (n)	14	8	− 6	.074
Use of rollator walker or walking aids during 6MWT (n)	9	4	− 5	.014*
Handgrip dominant hand (lbs)	38.8 ± 22.6	41.1 ± 22.9	2.3 ± 5.2	.073
Handgrip dominant hand (% of predicted)	60 ± 27	64 ± 24	4 ± 8	.064
Handgrip non-dominant hand (lbs)	32.8 ± 18.4	36.5 ± 19.7	3.7 ± 5.8	.014*
Handgrip non-dominant hand (% of predicted)	61 ± 25	68 ± 22	7 ± 10	.011*
Berg balance scale (points)	53.1 ± 3.2	54.1 ± 2.4	1 ± 1.6	.013*
Device-related echocardiography parameters				
Mitral valve insufficiency (grade)	0.98 ± 0.5	1.04 ± 0.5	0.06 ± 0.4	.503
Mean gradient mitral valve (mmHg)	3.07 ± 1.2	3.00 ± 1.3	− 0.07 ± 1.3	.849
Left ventricular ejection fraction (%)	47 ± 12	53 ± 13	6 ± 9	.013*

CR, cardiac rehabilitation; 6MWD, six-minute walking distance; 6MWT, six-minute walking test

During CR, exercise intensity started at a low intensity level and carefully increased over the course (p < 0.01). The mean 6MWD improved significantly from 272 ± 97 to 304 ± 111 m (p < 0.05), corresponding to an improvement from 60 ± 26% to 67 ± 29% of predicted values [19]. At the beginning of CR, 14 patients were below the critical prognostic threshold [20, 21] of 300 m, by the end only 8 patients. Dependence on a rollator walker or walking aids was significantly reduced from 9 to 4 patients (p < .05). Values of BBS improved significantly (p < .05), whereas handgrip strength values only improved for the non-dominant hand (p < .05). At the end of CR, no device-related problems were observed echocardiographically: no clip detachments, no changes regarding MI or related gradient, and even slightly improved left ventricular ejection fraction (p < .05).

At a socio-medical level, patients also profited: during CR, 4 patients could apply for household help and 3 patients for an official disability level.

## Discussion

### Feasibility and outcomes

To the best of our knowledge, this is the first published report on feasibility and outcomes of CR (whether performed inpatient or outpatient) immediately after percutaneous mitral valve reconstruction using clipping procedures. This is surprising since the positive effects of rehabilitation programs in other cardiac patient populations (e.g. coronary artery disease, HF, transcatheter aortic valve replacement, heart transplantation) are already well-known [12, 22] and CR should be recommended especially in older and frail patients [23].

One reason for a low level of CR utilization after MitraClip™ implantation could be that the risk of clip dislocation appears to be highest in the first period after implantation [24] and therefore physically rest is often recommended. As a consequence, it can be assumed that CR is rarely prescribed and appears to be underused in this patient population (even less than for “normal” HF patients [25]).

**Fig. 3:**
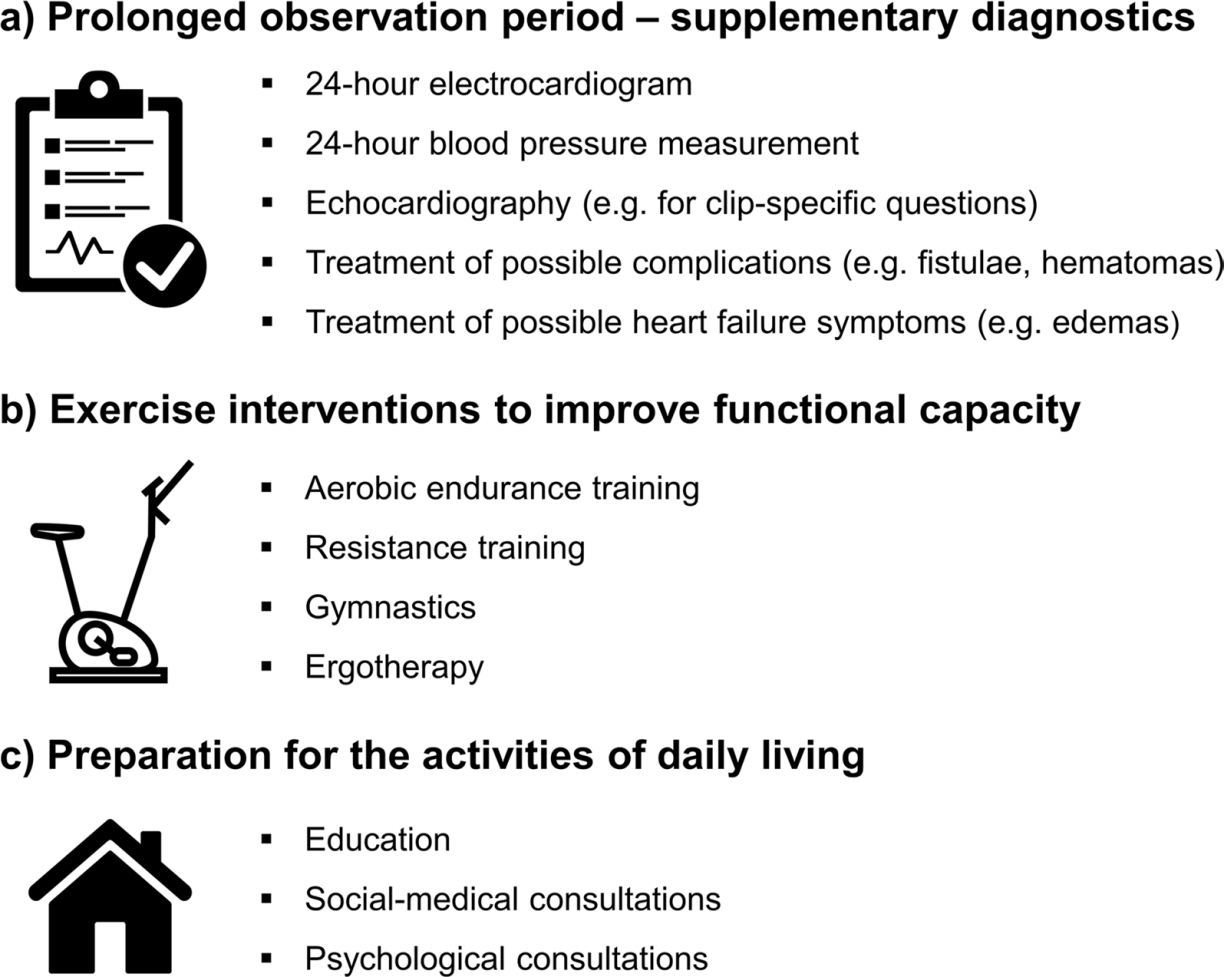
Potential benefits for participating in multimodal cardiac rehabilitation program after percutaneous mitral valve reconstruction using clipping procedures: **a** prolonged observation period and possibility for supplementary diagnostics, **b** individualised exercise interventions to improve functional capacity, **c** personalised preparation for activities of daily living

However, our experience shows that adapted inpatient CR in selected patients is definitively possible, and that patients can profit from multimodal CR at both a functional and a socio-medical level (Fig. 3). In our hospital, physical exercise training was purposely conducted at a low intensity level, with mean systolic blood pressure values of below 120 mmHg and maximum values of about 140 mmHg. The treatments led to no device-related complications, and functional capacity could be significantly improved during the rehabilitation period. Dependence on a rollator walker or walking aids could be reduced, indicating improved prerequisites for activities of daily living.

The 6MWD achieved of mean 304 (± 111) m was just above the critical prognostic threshold of 300 m [20] and the reached improvements during CR can be classified as moderate clinically important changes [26]. The observed functional capacity is approximately within the range measured in elderly patients undergoing CR after atrioventricular valve interventions [27] and in line with published MitraClip™ studies [28, 29]. Albeit compared with other special cardiological patient cohorts (e.g. PCI, transcatheter aortic valve replacement, left ventricular assist device implantation, heart transplantation), a reduced functional capacity must be assumed [27, 30, 31].

### Potential benefits of inpatient CR

#### Prolonged observation period

A main advantage of participating in an inpatient CR program after clip implantation is the significantly extended observation period (in Germany by 3 weeks) compared to a discharge directly home. During this time span supplementary diagnostic and therapies (e.g. 24-h ECG, 24-h blood pressure measurement, echocardiography, treatment of possible transcatheter complications, treatment of possible HF symptoms) can be performed in a clinical setting in order to adjust/optimize therapy and prevent complications that might go undetected/untreated at home [23]. Thus, in our study 4 patients (15%) with a worsening clinical condition could be identified and referred to acute HF department timely, with 1 patient able to continue inpatient CR after PCI.

#### Physical therapy and exercise interventions

The special needs of this often old and frail patient population can be addressed in an adequate manner by implementing individualized physical therapy and tailored exercise interventions during CR period [11, 23]. In addition to the treatment of possible orthopedic comorbidities, the achievement of sufficient coordinative and motoric functions should be aimed in order to prepare the patients for discharge home and the activities of daily living (e.g. balance training and fall prevention by physiotherapy and/or ergotherapy if needed). Patients are also introduced to moderate aerobic endurance and resistance training in a supervised clinical setting to counteract muscle deconditioning without causing blood pressure peaks.

#### Education, socio-medical care and psychological support

The transition from the clinic to the home environment is crucial and can often be accompanied by uncertainties, especially after a long hospital stay. With individual educational courses, socio-medical and psychological consultations, patients can be supported by specialized staff during CR period. The aim of these treatments is to prevent potential problems at home or to detect and eliminated them in time to prepare patients for the challenges of everyday life. Thus, in our study 4 patients (15%) were able to apply for household help at the health insurance companies and also 3 patients (11%) were able to apply for an official disability level at the local authority. These applications were all initiated and guided by socio-medical personnel during the CR period, and it is uncertain whether the applications would have been made without our external support.

### Study limitations and future investigastions

This is a retrospective pilot study with only a small sample size and without comparison of exercise values before and after implantation. Further studies are required to confirm and supplement our experience. These studies should systematically examine the special needs of this often frail patient population in order to develop specific rehabilitation programs. Likewise, prospective and controlled studies (e.g. CR vs, geriatric rehabilitation vs.no rehabilitation; inpatient vs. outpatient) would be highly desirable to examine the effect of CR on functional capacity, quality of life, readmissions and survival over the long-term course.

## Conclusions

The results of this study indicate that an adapted inpatient CR is feasible in selected patients after percutaneous mitral valve clipping procedure. MitraClip™ patients are usually a multimorbid patient group with many comorbidities (also orthopaedic) and a high need for care. However, our first results are encouraging: during therapeutic treatments no device-related complications occurred and functional capacity and dependence on rollator walker was significantly improved. Larger controlled studies should be aimed to improve evidence on exercise-related questions in MitraClip™ patients to allow for optimized recovery and aftercare after transcatheter mitral valve procedure.

## Data Availability

The datasets used and/or analysed during the current study are not publicly available due to our internal hospital policy but are available from the corresponding author on reasonable request.

## Abbreviations

BBS: Berg-Balance-Scale
CPET: Cardiopulmonary exercise test
CR: Cardiac rehabilitation
ECG: Electrocardiogram
HF: Heart failure
MI: Mitral insufficiency
PCI: Percutaneous coronary intervention
PMR: Physical medicine and rehabilitation
VHD: Valvular heart disease
6MWD: Six-minute walking distance
6MWT: Six-minute walking test
